# 53. Optimizing Transitions of Care Antimicrobial Prescribing at a Community Teaching Hospital

**DOI:** 10.1093/ofid/ofab466.255

**Published:** 2021-12-04

**Authors:** Kushal Naik, Jeremy J Frens, Jordan R Smith

**Affiliations:** 1 Cone Health, Greensboro, North Carolina; 2 High Point University, High Point, NC

## Abstract

**Background:**

Antimicrobial stewardship integral to patient care. Institutions with stewardship decrease antibiotic use, cost, and antibiotic-associated infections. However, few efforts have been formally made to address discharge antimicrobial prescribing, even though many patients started on antibiotic therapy in the hospital are prescribed oral antibiotics to complete their regimens.

**Methods:**

This was an IRB approved, quasi-experimental, pre-post study. Patients were included if they were >18 years and were discharged from the hospital with an oral antibiotic prescription. Patients discharged against medical advice, prescribed indefinite prophylactic antimicrobial therapy for legitimate reasons, or discharged to a skilled nursing facility were excluded. The retrospective group evaluated a random sample of patients discharged in 2/2020. The prospective group included patients discharged between 1/2021 – 6/2021. In the prospective group, a clinical pharmacist assessed the indication for antibiotics and pended discharge antibiotic prescriptions for physician review. Antibiotic choice and duration of therapy were based on local and national guidelines.

Patient Screening for Inclusion and Exclusion

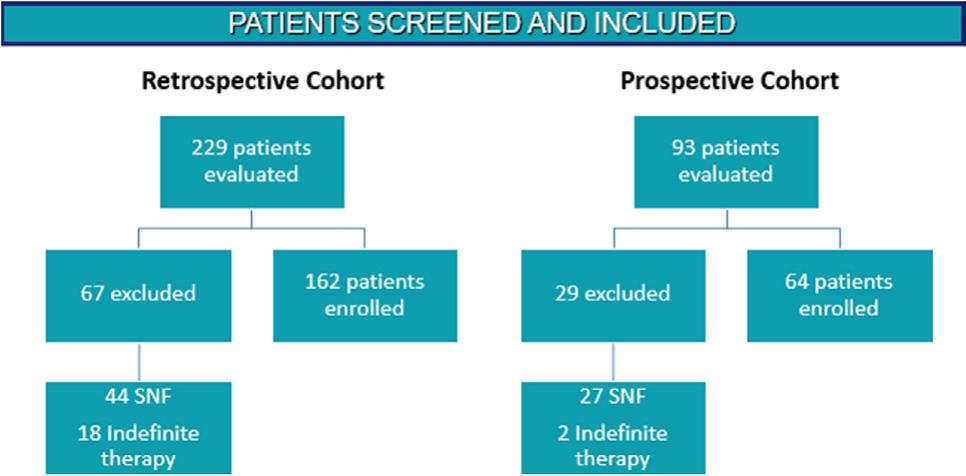

Breakdown of patients screened, included, and excluded for study

**Results:**

86 (53.1%) of 162 retrospective patients from 2/2020 prior to implementation of the program demonstrated were discharged on inappropriate antimicrobial therapy with excessive duration being the principal driver for inappropriateness. In the prospective group of 64 patients, the rate of patients discharged on inappropriate antibiotics decreased to 28.1% (p=0.001). The duration of inappropriate therapy decreased from a mean of 4.6 days to 2.7 days (p=0.001). 45 (70.3%) of 64 prospective pharmacist’s interventions were accepted by providers.

Study Outcomes

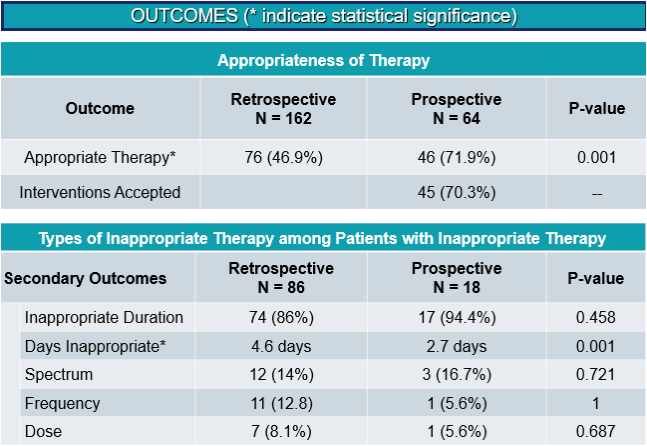

Outcomes including overall appropriate prescribing, appropriate duration, spectrum, frequency, and dose, as well as days of inappropriate therapy

**Conclusion:**

Literature demonstrates that prospective evaluation of discharge antibiotics by a clinical pharmacist is effective in improving appropriateness of discharge antibiotic prescriptions, optimizing duration of outpatient antibiotics as well as reducing unnecessarily broad-spectrum therapy. The prospective results from this study demonstrate that this innovative approach can improve outpatient oral antibiotic prescribing and provide a framework for other institutions to implement similar programs.

**Disclosures:**

**All Authors**: No reported disclosures

